# Soil-transmitted helminthiasis in four districts in Bangladesh: household cluster surveys of prevalence and intervention status

**DOI:** 10.1186/s12889-020-08755-w

**Published:** 2020-05-12

**Authors:** Stacy L. Davlin, Alexander H. Jones, Sanya Tahmina, Abdullah Al Kawsar, Anand Joshi, Sazid I. Zaman, Muhammad M. Rahman, Bozena M. Morawski, Michael S. Deming, Rubina Imtiaz, Mohammad J. Karim

**Affiliations:** 1 Children Without Worms, The Task Force for Global Health, 325 Swanton Way, Decatur, GA USA; 2 Communicable Disease Control Department, Directorate General of Health Services, Ministry of Health and Family Welfare, Road # 29, New DOHS, Mohakhali, Dhaka, Bangladesh; 3 Elimination of Lymphatic Filariasis & STH Control Program, Directorate General of Health Services, Ministry of Health & Family Welfare, Road # 29, New DOHS, Mohakhali, Dhaka, Bangladesh; 4 Mahidol Oxford Tropical Medicine Research Unit, Faculty of Tropical Medicine, Mahidol University, C/O DOHS, Road # 29, New DOHS, Mohakhali, Dhaka, Bangladesh

**Keywords:** Soil-transmitted helminthiasis, Epidemiology, Integrated survey, Preventive chemotherapy, Water, sanitation, and hygiene, Bangladesh

## Abstract

**Background:**

In 2016, after 8 years of twice-annual nationwide preventive chemotherapy (PC) administration to school-age children (SAC), the Bangladesh Ministry of Health & Family Welfare (MOHFW) sought improved impact and intervention monitoring data to assess progress toward the newly adopted goal of eliminating soil-transmitted helminthiasis (STH) as a public health problem.

**Methods:**

We surveyed four Bangladeshi districts between August and October 2017. We conducted a multi-stage, cluster-sample, household survey which produced equal-probability samples for preschool-age children (PSAC; 1–4 years), SAC (5–14 years), and adults (≥ 15 years). Standardized questionnaires were administered, using Android-based smart phones running an Open Data Kit application. Stool samples were collected and testing for STH prevalence and infection intensity used the Kato-Katz technique.

**Results:**

In all, 4318 stool samples were collected from 7164 participants. Estimates of STH prevalence by risk group in three of the four surveyed districts ranged from 3.4 to 5.0%, all with upper, 1-sided 95% confidence limits < 10%. However, STH prevalence estimates in Sirajganj District ranged from 23.4 to 29.1%. Infections in that district were spatially focal; four of the 30 survey clusters had > 50% prevalence in at least one risk group. Among all tested specimens, *Ascaris lumbricoides* was the most common STH parasite [8.2% (*n* = 352)], followed by *Trichuris trichiura* [0.9% (*n* = 37)], and hookworm [0.6% (*n* = 27)]. In each district, PC coverage among SAC was above the 75% program target but did not exceed 45% among PSAC in any district. Improved sanitation at home, school, or work was over 90% in all districts.

**Conclusions:**

In the three low-prevalence districts, the MOHFW is considering decreasing the frequency of mass drug administration, per World Health Organization (WHO) guidelines. Also, the MOHFW will focus programmatic resources and supervisory efforts on Sirajganj District. Despite considering WHO guidance, the MOHFW will not expand PC administration to women of reproductive age partly due to the low prevalence of hookworm and *T. trichiura*, the STH parasites that contribute most to morbidity in that risk group. Data collected from surveys such as ours would help effectively guide future STH control efforts in Bangladesh and elsewhere.

## Background

Over one billion people in more than 100 countries have soil-transmitted helminthiasis (STH) [1] which is caused by the parasites *Ascaris lumbricoides*, *Trichuris trichiura*, and the hookworm species, *Necator americanus* and *Ancylostoma duodenale*. The World Health Organization (WHO) has established the goal of elimination of STH as a public health problem by 2020, defined as < 1% moderate-to-high intensity infection (MHII) in at-risk preschool-age children (PSAC; 1–4 years old) and school-age children (SAC; 5–14 years old) [2]. Women of reproductive age (WRA; 15–49 years old) are another WHO-identified risk group [3].

Many national STH control programs, including in Bangladesh, have struggled to demonstrate progress toward elimination of STH as a public health problem. Additionally, national programs often rely on school-based preventive chemotherapy (PC) targeting SAC as the primary intervention to achieve the program goal. PSAC and WRA receive PC through different platforms, if at all. Bangladesh and other national programs use PC coverage as the primary indicator of success. While important, PC coverage does not directly evaluate impact or the prevalence thresholds used to determine mass drug administration (MDA) frequency [4]. Additionally, many national programs rely on the WHO methodology for school-based parasitologic surveys to monitor prevalence [5]. Given species-specific epidemiology and drug sensitivity, parasitologic assessment of SAC alone may not accurately represent STH epidemiology in all risk groups.

In 2005 and 2007, the Bangladesh Ministry of Health & Family Welfare (MOHFW) conducted baseline school-based STH parasitologic surveys [5] in 5 of 64 districts and found that 80.0% of surveyed school children were STH positive. Consequently, the MOHFW initiated a nationwide STH control program in 2008 and has since conducted twice-annual MDA among SAC. By April 2018, the program completed 20 nationwide rounds of MDA using mebendazole. Additionally, 19 districts received annual community-wide MDA for lymphatic filariasis (LF). Since 2013, the MOHFW has reported > 75% annual national PC coverage for SAC [6] and currently targets 40.6 million children. The MOHFW adopted the goal of eliminating STH as a public health problem among children in 2016, which necessitated increased program monitoring. To assess progress toward the newly adopted goal, the MOHFW and Children Without Worms developed the “Integrated Community-based Survey for Program Monitoring” (ICSPM).

Based on a standardized protocol, the ICSPM provides statistically valid, district-level estimates of STH and parasite-specific prevalence and intensity of infection for each risk group; evaluates MOHFW-reported PC coverage; and measures sanitation coverage and specific hygiene-related behaviors. This publication presents the results of four ICSPM surveys and discusses their programmatic implications. There are no baseline data from the surveyed districts. Further evaluation of the ICSPM methodology is planned with the hope that the methodology will benefit other national programs that have the same programmatic goal.

## Methods

### Study setting and population

**Fig. 1:**
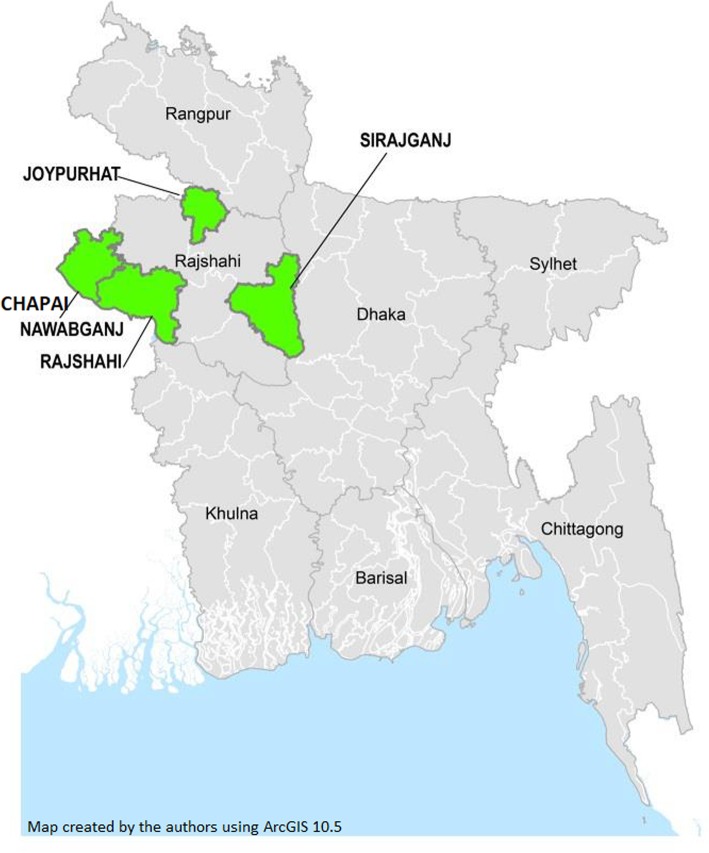
Districts surveyed, Rajshahi Division, Bangladesh, August – October 2017

We surveyed Joypurhat, Chapai Nawabganj, Sirajganj, and Rajshahi districts (Fig. 1). Joypurhat has never received LF interventions. Chapai Nawabganj, Sirajganj, and Rajshahi districts received multiple years of LF MDA before stopping in 2013, 2012, and 2011, respectively [7]. District population size ranges from 1.0 million (Joypurhat) to 3.1 million (Sirajganj). The population is predominantly rural (range from 69% to 86%). The districts were selected because they were participating in a joint STH-nutrition project, which required baseline assessment. We sampled the WHO risk groups: PSAC (1–4 years old) and SAC (5–14 years old). The MOHFW also decided to sample all adults (≥ 15 years old).

### Ethical considerations

The survey protocol was approved by the Bangladesh Medical Research Council. Participation in the survey was voluntary. Informed consent was obtained from all participants ≥ 18 years old. Enrollment of participants < 18 years old required consent from a parent or guardian and assent from any person 5–17 years old. Participants 1–4 years old were enrolled with consent of a parent or guardian.

### Study design and data collection

The ICSPM is a multi-stage, cluster-sample household survey. It expands the WHO *Assessing the epidemiology of STH during a TAS module* [4] by including all WHO-identified STH risk groups rather than SAC only. It modifies the sampling design of the WHO *Transmission Assessment Survey for LF Elimination* by adding segmentation, which improves efficiency of household selection in the field [8].

The ICSPM facilitates classification of each STH risk group according to WHO-defined prevalence ranges [5]. Risk group prevalence is classified by calculating the upper, 1-sided 95% confidence limit. The prevalence range in which this limit falls is the lowest range in which there can be 95% confidence that true prevalence lies. The target sample size of 332 persons per risk group is adequate for determining if a < 10% prevalence threshold has been achieved and for higher thresholds. Details of this approach are discussed elsewhere [9].

The sample design provides an equal-probability sample from each risk group in each district. Villages were used as primary sampling units as data on census enumeration areas were unavailable. The number of households in a village was estimated based on 2011 national census data adjusted for population growth [10]. In all, 30 villages (clusters) were selected using probability proportional to their size in 100-household segments. In the field, these villages were subdivided into segments of approximately equal size. One segment was randomly selected, and households within it were systematically selected at a rate estimated to provide the needed sample size for the least populous risk group (PSAC). In households already selected for PSAC, SAC and adults were eligible for the survey sample. All members of the indicated risk-group(s) in a household were eligible.

Trained survey teams used Android-based smart phones running an Open Data Kit application to administer standardized survey questionnaires and collect geographic coordinates at each selected household. Data collection occurred between August and October 2017, approximately 5 months after the last STH MDA and approximately 1 month before the next one. During data collection, data issues were identified and addressed daily.

### Sample collection and laboratory procedures

Plastic stool containers, without preservative, were left at selected households overnight and collected the next morning. Survey teams instructed participants on sample collection, emphasizing depositing stool samples the morning of collection. After collection, samples were stored in coolers and transported to field laboratories within 2 h. Upon arrival, medical technologists processed samples by conducting dual-slide microscopy using the Kato-Katz technique [11]. A laboratory supervisor consulted on discordant results and independently reviewed 10% of samples.

### Data analysis

De-identified data were stored on a secure cloud-based platform and analyzed using Stata version 15 (StataCorp. 2017. Stata Statistical Software: Release 15. College Station, TX) while accounting for the cluster sample survey design. All estimates include two-sided 95% confidence intervals except for district-level prevalence which are designed to show the lowest WHO threshold achieved by calculating the upper, one-sided 95% confidence limit. A Chi-square (χ^2^) test was used to test for significant differences in prevalence. *P*-values were considered significant if < 0.05. Intensity of infection was determined according to WHO-defined classifications (Table 1). Missing data were typically addressed during data collection; however, if data were missing after data collection, they were excluded from analysis.

**Table 1:** WHO classifications of STH infection intensity (eggs per gram of stool) [2]

Organism	***Light intensity infection***	***Moderate intensity infection***	***Heavy intensity infection***
***A. lumbricoides***	< 5000	5000 - 49,999	≥ 50,000
***T. trichiura***	< 1000	1000 - 9999	≥ 10,000
**Hookworm**	< 2000	2000 - 3999	≥ 4000

The authors generated all maps using ArcGIS 10.5 (Environmental Systems Research Institute, Redlands, CA). A central location within the cluster was selected for representation on maps.

## Results

### Enrollment and sampling rates

In the four surveyed districts, 7164 participants were enrolled and completed the questionnaire. Among those enrolled, 4361 (61%) were female. Ages ranged from 1 to 80 years. A total of 4321 (60.3%) provided a stool sample (Table 2), and among those, 4318 provided enough stool for testing. Insufficient training and supervision led to a failure to record the number of eligible participants within each household that were either unwilling or unable to participate; however, the overall non-response rate among enrolled persons was 39.7% (*n* = 2843). It was 42.3% among PSAC, 40.2% among SAC, and 36.0% among adults. Among adults, males constituted only 10% of those enrolled and 8.5% of those who provided a sample. Adult males were frequently unavailable during the daytime when survey teams were present.

**Table 2:** Enrollment and sampling rates among PSAC, SAC, and adults by district

	PSAC (1–4 years) ***n = 2448***		SAC (5–14 years) ***n = 2614***		Adults (≥ 15 years) ***n = 2102***		Total ***N = 7164***	
	Enrolled^**a**^	Provided Sample	Enrolled^**a**^	Provided Sample	Enrolled^**a**^	Provided Sample	Enrolled^**a**^	Provided Sample
District	*n*	*n* (%)	*n*	*n* (%)	*n*	*n* (%)	*n*	*n* (%)
Chapai Nawabganj								
Male	318	170 (53.5)	357	207 (58.0)	29	14 (48.3)	704	391 (55.5)
Female	303	175 (57.8)	329	190 (57.8)	451	299 (66.3)	1083	664 (61.3)
Joypurhat								
Male	281	190 (67.6)	306	179 (58.5)	120	68 (56.7)	707	437 (61.8)
Female	250	138 (55.2)	329	196 (59.6)	462	300 (64.9)	1041	634 (60.9)
Rajshahi								
Male	352	201 (57.1)	308	204 (66.2)	29	17 (58.6)	689	422 (61.2)
Female	285	168 (58.9)	320	179 (55.9)	496	314 (63.3)	1101	661 (60.0)
Sirajganj								
Male	342	205 (59.9)	328	201 (61.3)	33	15 (45.5)	703	421 (59.9)
Female	317	166 (52.4)	337	206 (61.1)	482	319 (66.2)	1136	691 (60.8)

^a^Number of participants who consented and answered questionnaire

### STH prevalence

**Fig. 2:**
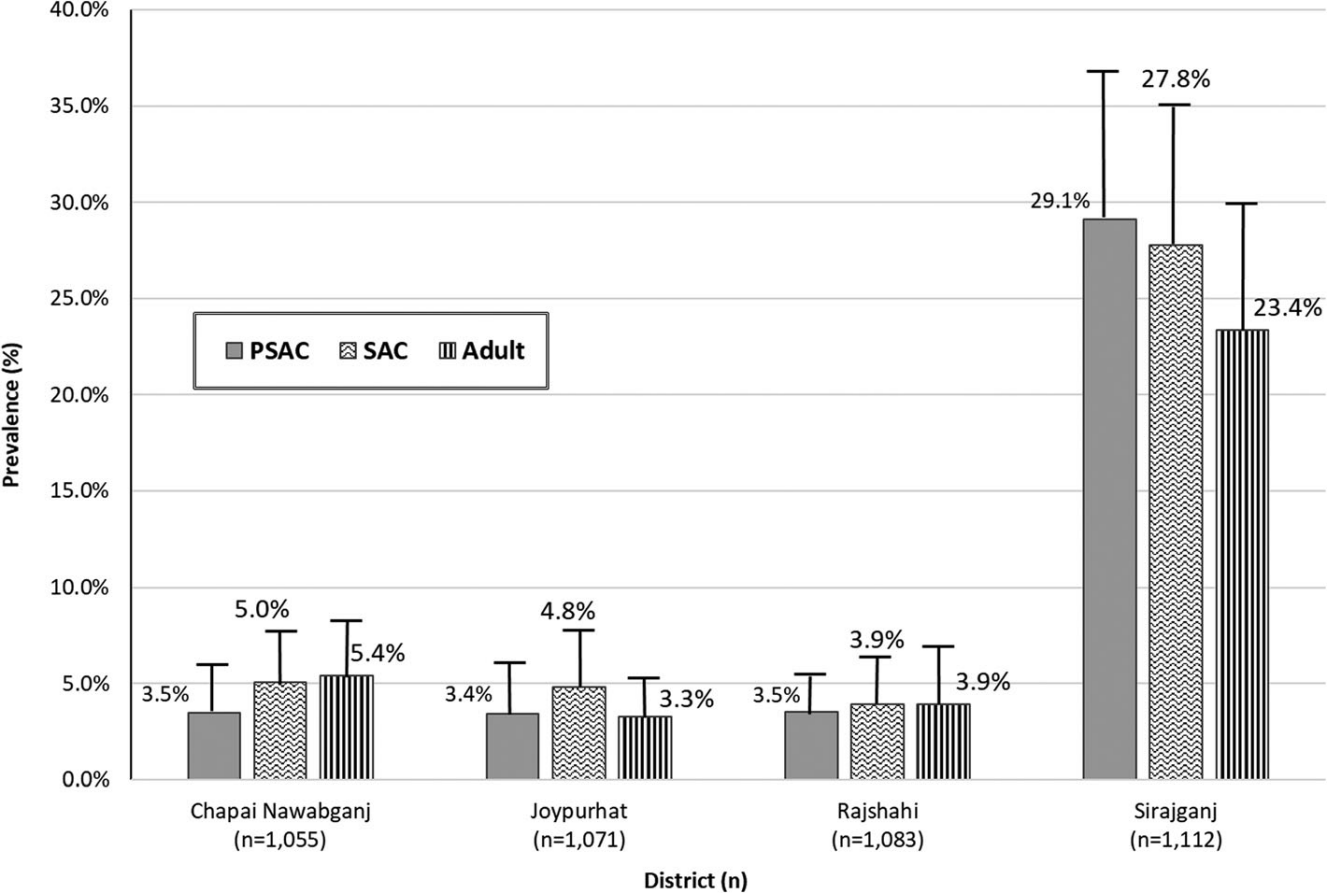
Prevalence (upper, one-sided, 95% confidence limit) of any STH by risk group and district

The upper, one-sided 95% confidence limit for prevalence of any STH was < 10% among all risk groups in Joypurhat, Chapai Nawabganj, and Rajshahi districts. The STH point prevalence in each risk group was significantly higher (*p* < 0.001) in Sirajganj District than other districts. District-level STH prevalence ranged from 3.3% (upper, one-sided, 95% confidence limit = 5.0%) among adults in Joypurhat District to 29.1% (upper, one-sided, 95% confidence limit = 36.0%) among PSAC in Sirajganj District (Fig. 2).

Risk group-specific STH prevalence estimates were similar across Chapai Nawabganj, Joypurhat, and Rajshahi districts ranging from 3.3 to 5.4%. Risk group prevalence in Sirajganj District ranged from 23.4 to 29.1% and was significantly higher than in other districts (all p-values < 0.001).

### Moderate-to-high intensity infection

**Fig. 3:**
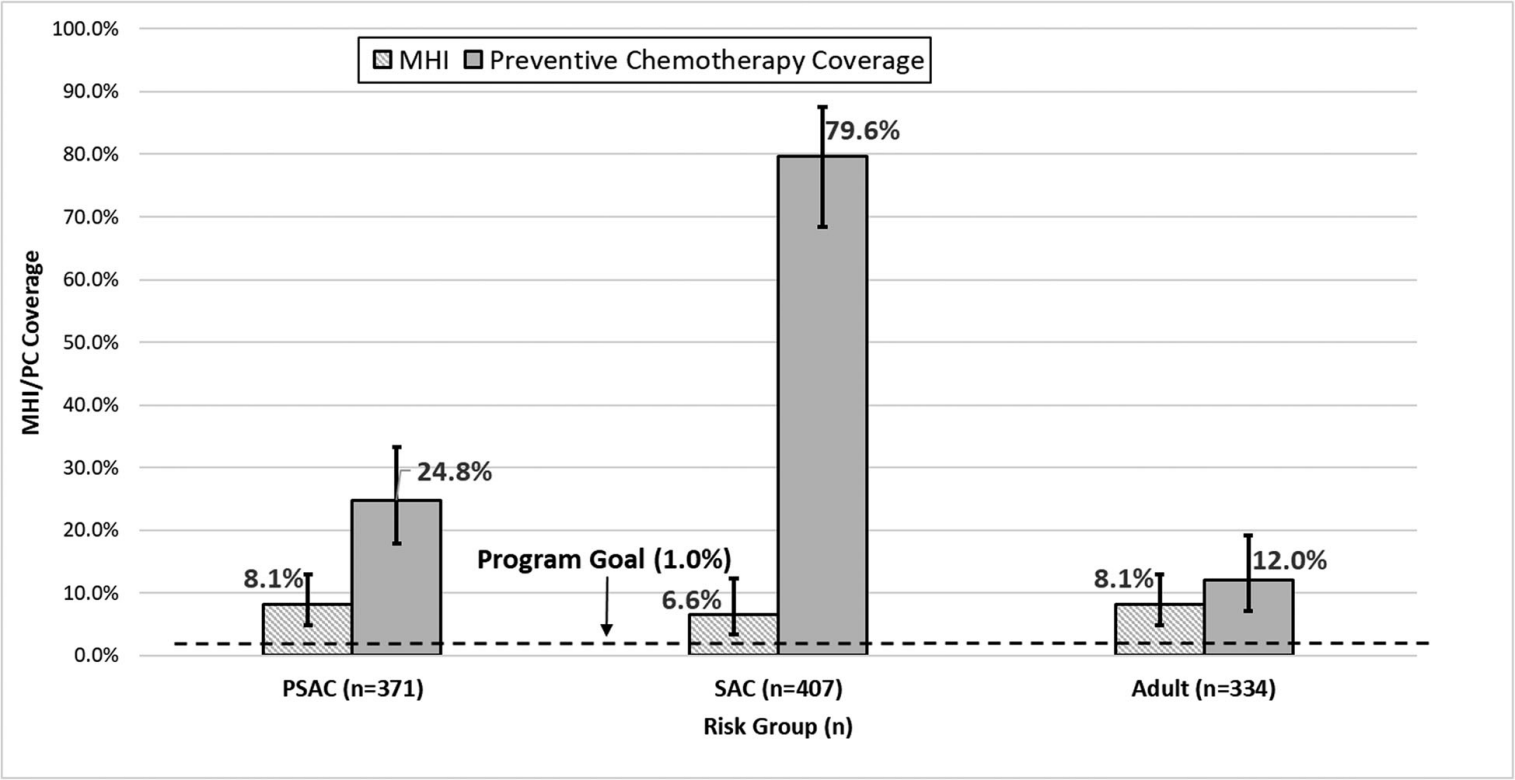
Any STH MHII and PC coverage (95% CI) by risk group, Sirajganj District

Chapai Nawabganj and Joypurhat districts each had one participant with MHII. No MHII was detected in Rajshahi District. In Sirajganj District, MHII was 8.1% (95% CI: 4.8–13.2%), 6.6% (95% CI: 3.4–12.7%), and 8.1% (95% CI, 4.4–14.4%) among PSAC, SAC, and adults respectively (Fig. 3). Risk group-specific MHII estimates in Sirajganj District were significantly higher than other districts (all p-values < 0.001) and well above the threshold for elimination as a public health problem (< 1%). The percentage of MHII and PC coverage by risk group are shown for Sirajganj District in Fig. 3.

### Parasite type

**Fig. 4:**
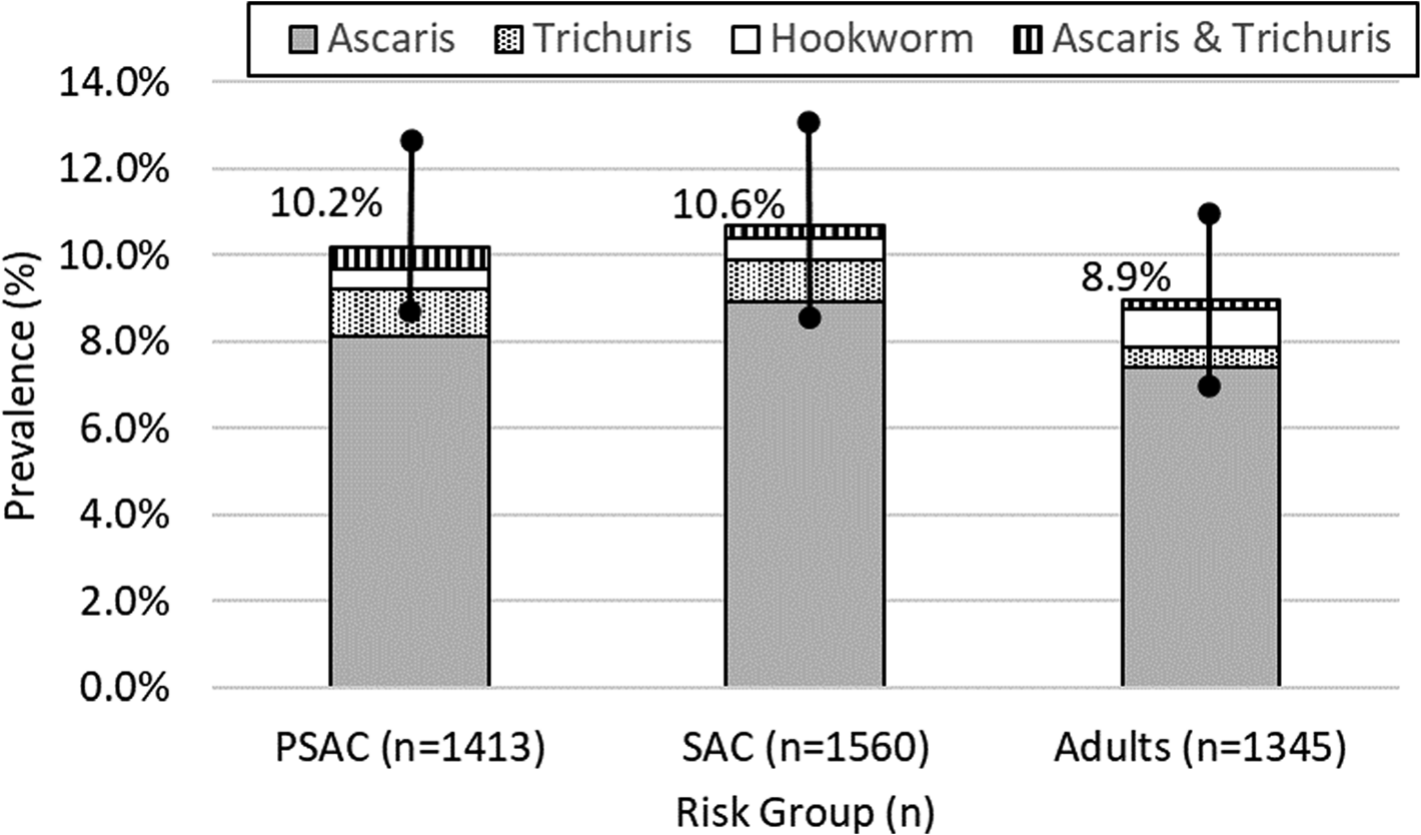
STH prevalence (95% CI) by parasite type and risk group in all districts

In all districts, *A. lumbricoides* was the most common STH parasite [8.2% (*n* = 352)], followed by *T. trichiura* [0.9% (*n* = 37)] and hookworm [0.6% (*n* = 27)]. *A. lumbricoides* was most prevalent among SAC [8.9% (*n* = 139)], followed by PSAC [8.1% (*n* = 114)] and adults [7.4% (*n* = 99)]. All polyparasitism was due to *A. lumbricoides* and *T. trichiura* [0.3% (*n* = 14)] and only identified in Sirajganj District. Prevalence of each species was consistent across risk groups (Fig. 4). Hookworm was the least common infection (adults *n* = 12, SAC *n* = 7, PSAC *n* = 8). No significant differences in STH parasite type were observed across districts.

### Geographic distribution of STH

**Fig. 5:**
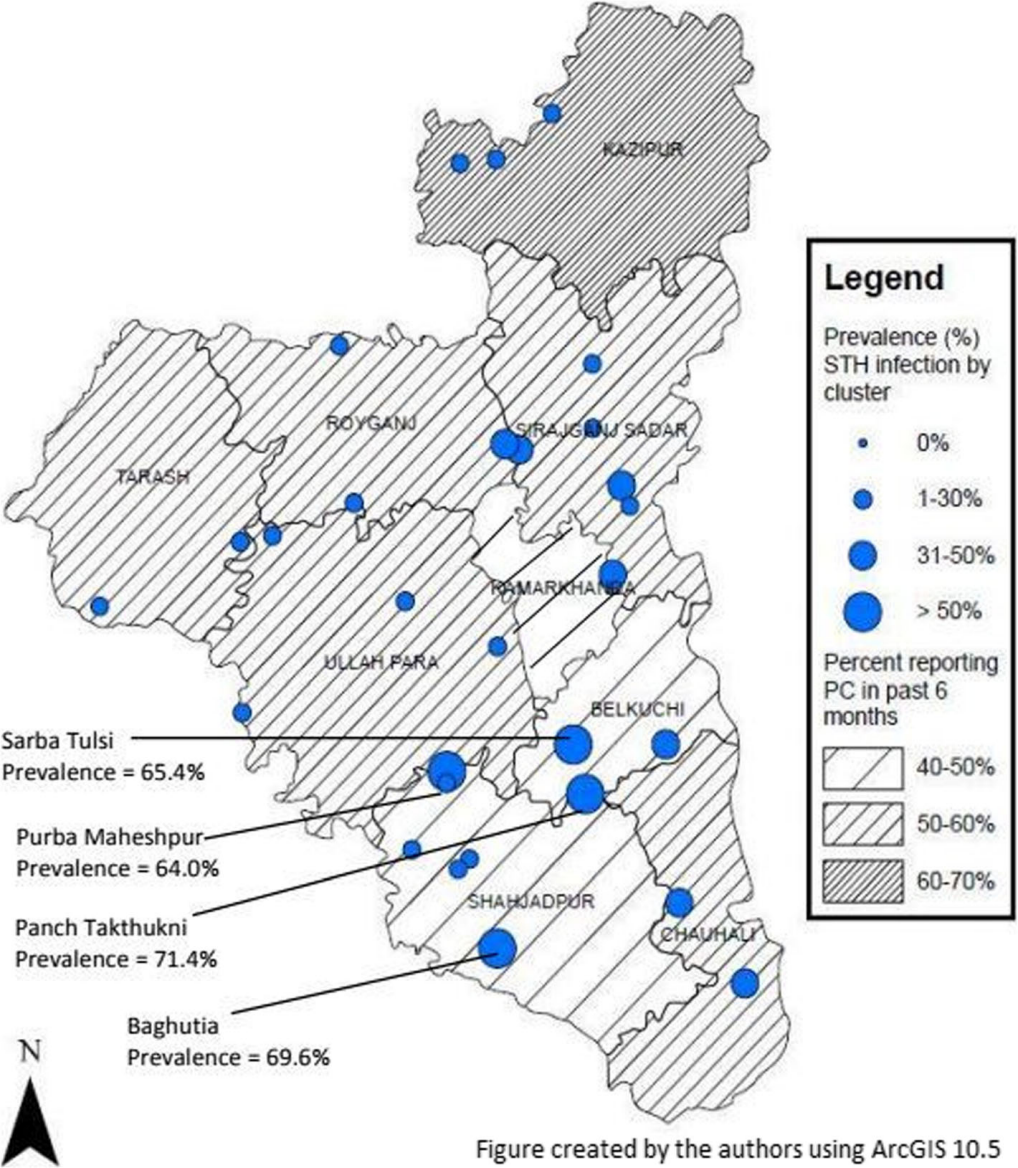
Geographic distribution of STH prevalence and PC coverage in all risk groups combined, Sirajganj District

Within and across districts, the geographic distribution of STH prevalence varied. Due to the geographic distribution and high prevalence in Sirajganj District, we mapped overall prevalence and PC coverage (Fig. 5). Sirajganj sub-districts of concern included Belkuchi (SAC: 57.1% prevalence, 20.0% MHII) and Kamarkhanda (SAC: 50.0% STH prevalence, 28.6% MHII). Belkuchi had the highest STH prevalence in PSAC [60.5% (*n* = 23)], SAC [57.1% (*n* = 20)], and adults [50.0% (*n* = 15)] (Table 3). Belkuchi had the highest MHII among PSAC [23.7% (*n* = 9)] and adults [20.0% (*n* = 6)], and Kamarkhanda had the highest MHII among SAC. Three sub-districts had no MHII (Chauhali, Kazipur, and Tarash) and STH prevalence in those sub-districts ranged from 3.2% (n = 1) among adults in Kazipur to 42.9% (n = 9) among SAC in Chauhali.

**Table 3:** STH prevalence^a^ and PC coverage (95% CI) by risk group and sub-district^b^, Sirajganj District

Sub-district (risk group = n)	***PSAC***				***SAC***				***Adults***			
	***Any STH***		***PC received ≤ 6 months ago***		***Any STH***		***PC received ≤ 6 months ago***		***Any STH***		***PC received ≤ 6 months ago***	
	***n***	% (95% CI)	***n***	% (95% CI)	***n***	% (95% CI)	***n***	% (95% CI)	***n***	% (95% CI)	***n***	% (95% CI)
Belkuchi PSAC = 38;SAC = 35;Adult = 30	23	60.5 (39.4–78.3)	14	36.8 (19.9–57.8)	20	57.1 (39.5–73.1)	21	60.0 (17.6–91.4)	15	50.0 (20.2–79.8)	11	36.7 (20.0–57.2)
Chauhali PSAC = 18; SAC = 21; Adult = 18	6	33.3 (23.4–44.9)	1	5.6 (0.5–39.9)	9	42.9 (37.0–48.9)	20	95.2 (86.0–98.5)	5	27.8 (15.7–44.2)	0	0.0 N/A
Kamarkhanda PSAC = 18; SAC = 14; Adult = 11	5	27.8 ^c^	3	16.7 ^c^	7	50.0 ^c^	11	78.6 (78.6–78.6)	1	9.1 ^c^	1	9.1 ^c^
Kazipur PSAC = 48; SAC = 39; Adult = 31	3	6.3 (1.8–19.3)	20	41.7 (12.3–78.4)	2	5.1 (2. –11.7)	33	84.6 (54.3–96.2)	1	3.2 (0.5–19.3)	6	19.4 (4.1–57.6)
Royganj PSAC = 38; SAC = 41; Adult = 37	8	21.1 (13.9–30.6)	11	28.9 (15.7–47.1)	11	26.8 (14.2–44.8)	33	80.5 (44.5–95.5)	4	10.8 (6.5–17.5)	5	13.5 (8.3–21.2)
Shahjadpur PSAC = 66; SAC = 65; Adult = 68	23	34.8 (20.9–51.9)	14	21.2 (9.5–40.9)	15	23.1 (8.7–48.6)	50	76.9 (54.6–90.2)	19	27.9 (19.9–37.6)	3	4.4 (1.2–15.1)
Sirajganj Sadar PSAC = 65; SAC = 81; Adult = 57	23	35.4 (24.9–47.5)	13	20.0 (10.5–34.7)	24	29.6 (18.0–44.6)	64	79.0 (46.6–94.2)	14	24.6 (16.0–35.8)	8	14.0 (4.4–36.9)
Tarash PSAC = 23; SAC = 28; Adult = 22	2	8.7 (2.3–27.9)	2	8.7 (2.3–27.9)	2	7.1 (1.6–27.3)	28	100.0 N/A	2	9.1 (1.9–33.5)	2	9.1 (1.9–33.5)
Ullah Para PSAC = 57; SAC = 83; Adult = 60	15	26.3 (12.9–46.2)	14	24.6 (11.7–44.5)	23	27.7 (14. –45.9)	64	77.1 (44.0–93.5)	17	28.3 (17.2–42.9)	4	6.7 (2.4–17.3)

^a^Among participants who provided a valid stool sample

^b^Sample size was powered to the district-level only and estimates are less precise and stable at the sub-district level because of the smaller number of observations and clusters

^c^95% confidence intervals not calculated due to only one strata and small number of observations

### Preventive chemotherapy coverage and water, sanitation and hygiene (WASH) indicators

In all districts, self-reported ICSPM PC coverage for SAC was above the MOHFW target of ≥ 75% [2] (range from 79.6 to 89.8%). However, ICSPM PC coverage was significantly lower in PSAC [overall = 32.6% (p < 0.001)] and adults [overall = 19.5% (p < 0.001)] than in SAC.

We also compared ICSPM PC coverage among SAC to SAC coverage reported by the MOHFW as part of routine program monitoring. MOHFW-reported coverage was from the MDA preceding the ICSPM [MOHFW unpublished data, 2017 (data not shown)]. Reported coverage data for other risk groups were unavailable. ICSPM PC coverage in SAC was lower, in some districts substantially, than MOHFW-reported coverage. ICSPM and reported coverage differed noticeably in Chapai Nawabganj (80.6 and 100%, respectively) and Sirajganj District (79.6 and 95.5%, respectively). ICSPM results across districts showed the most common source of deworming drugs for SAC was school-based MDA, while purchasing PC was the most common source for PSAC and adults (Table 4).

**Table 4:** Preventive chemotherapy coverage and most common source of PC (95% CI) by risk group and district

	***PSAC***		***SAC***		***Adults***	
	*n*	% [95% CI]	*n*	% [95% CI]	*n*	% [95% CI]
**District**	***PC received ≤ 6 months ago***					
*Chapai Nawabganj*	152	44.1 [35.1–53.4]	320	80.6 [69.3–88.4]	65	20.8 [15.3–27.5]
*Joypurhat*	112	34.1 [26.2–43.1]	324	86.3 [77.8–91.9]	93	25.3 [20.0–31.5]
*Rajshahi*	104	28.2 [20.0–38.1]	344	89.8 [82.2–94.4]	64	19.3 [13.0–27.8]
*Sirajganj*	92	24.8 [17.9–33.3]	324	79.6 [68.4–87.6]	40	12.0 [7.2–19.2]
**District**	***Most common source of PC***^***a***^					
*Chapai Nawabganj*	192	Purchased 86.1 [78.8–91.1]	347	School 90.1 [84.6–93.8]	150	Purchased 80.2 [71.7–86.6]
*Joypurhat*	110	Purchased 60.8 [45.3–74.3]	319	School 89.0 [83.3–93.0]	182	Purchased 83.9 [73.7–90.6]
*Rajshahi*	146	Purchased 80.2 [71.4–86.8]	353	School 93.4 [88.4–96.3]	168	Purchased 74.3 [62.1–83.7]
*Sirajganj*	177	Purchased 94.7 [88.7–97.6]	324	School 82.4 [73.0–89.1]	190	Purchased 90.9 [82.0–95.6]

^a^Survey question: “The last time you swallowed deworming medication, where did you receive it?”

Improved sanitation at home, school, or work was over 90% in all districts. The prevalence of improved flooring ranged from 10.6% (95% CI: 8.9–12.6%) in Sirajganj District to 34.7% (95% CI: 24.5–46.5%) in Chapai Nawabganj District. The presence of a handwashing station with soap in the home was observed in < 50% of households in all districts. Sirajganj District had the lowest observed household coverage of handwashing stations with soap (36.2%; 95% CI: 21.1–54.7%); however, compared to the other districts the difference was not statistically significant (Table 5).

**Table 5:** Sanitation and hygiene indicators (95% CI) by district

	***Chapai Nawabganj*** ***n = 1055***	***Joypurhat*** ***n = 1068***	***Rajshahi*** ***n = 1083***	***Sirajganj*** ***n = 1112***
**WASH Indicator**	**% [95% CI]**	**% [95% CI]**	**% [95% CI]**	**% [95% CI]**
*Improved sanitation*^*a*^*(home)*	97.6 [94.4–99.0]	94.0 [84.7–97.8]	98.3 [94.7–99.5]	95.3 [89.6–98.0]
*Handwashing station with soap (home)*	42.3 [26.4–60.0]	42.6 [26.8–60.1]	44.5 [28.6–61.6]	36.2 [21.1–54.7]

^a^Improved sanitation defined as: flush toilet to sewer/septic; ventilated improved pit latrine; pit latrine w/ slab; or composting toilet

## Discussion

Comprehensive, community-based surveys such as the ICSPM are intended to support STH program monitoring, particularly for advanced programs that have delivered interventions for ≥ 5 years. WHO recommends that programs assess, using prevalence ranges, possible changes to MDA frequency following 5–6 years of MDAs [5]. Given ICSPM results on prevalence and PC and sanitation coverage, as well as 20 completed MDA rounds, the MOHFW will pilot stopping the twice-annual STH MDAs in selected areas within Joypurhat, Rajshahi, and Chapai Nawabganj districts. WHO recommends MDAs once every 2 years in areas with ≥ 2% and < 10% prevalence [4]. However, the MOHFW is cautiously approaching the decision given potential disease recrudescence and the lack of established surveillance for STH. Other national programs likely have similar concerns. Further guidance to national programs on monitoring STH in low-prevalence districts is warranted, including on methods for parasitologic assessments or surveillance if MDAs cease. Outside of pilot areas, the MOHFW will continue twice-annual MDAs. The MOHFW will consider sanitation coverage in selecting pilot areas. Although MOHFW does not deliver WASH interventions, WASH partners could use ICSPM data to geographically target interventions. Additionally, the low prevalence of improved household flooring (10.6%) and of home handwashing stations with soap (36.2%) highlights the need for behavioral interventions in Sirajganj District.

Joypurhat, Rajshahi, and Chapai Nawabganj districts are approaching elimination of STH as a public health problem, with MHII across risk groups of < 10%. However, the MOHFW does not currently plan to assess whether the 1% “elimination” target has been met. The high cost required to obtain an adequate survey sample size is currently prohibitive. Furthermore, existing WHO guidance uses prevalence of any STH instead of MHII to determine MDA frequency. Limited available resources could be better used given the lack of a clear action tied to reaching the “elimination” target.

Similar to our results, high STH prevalence has been noted to be spatially focalized in Kenya [12], Burkina Faso [13], and Honduras [14]. Additionally, a recent systematic review in Asia found substantial heterogeneity in the geographic distribution of STH between and within countries [15]. Targeted programmatic focus by the MOHFW on high prevalence areas should improve overall intervention efficiency and impact. ICSPM results identified Sirajganj District for such enhanced control efforts. In Sirajganj, low PC coverage, particularly among PSAC (24.8%), likely contributes to high STH prevalence and MHII. Among PSAC who received PC in the district, 94.7% relied on purchasing the drug, indicating the need to improve MOHFW efforts to reach this risk group. Bangladesh has experienced inconsistent drug availability for PSAC partly due to procurement challenges. MDAs for SAC use drug donated through WHO, while there is no available donation for PSAC. Regardless of the impressive PC coverage of SAC, the MOHFW is unlikely to eliminate STH as a public health problem with the generally low coverage of PSAC. In surveyed districts, 44.1% was the highest district-level PSAC coverage.

The discrepancy between self-reported ICSPM SAC PC coverage and MOHFW-reported SAC PC coverage in Sirajganj District (79.6%. and 95.5%, respectively) suggests over-reporting in routine MOHFW monitoring of MDAs, hindering the timely addressing of implementation challenges. Recall bias could contribute to differing coverage figures. Additional PC coverage assessments will benefit the program, and enhanced MOHFW supervision during MDAs will be a priority going forward.

In Sirajganj, enhanced MOHFW supervision will further MOHFW efforts to identify and address programmatic gaps. Increased supervision, from national, division, and district personnel, will build capacity of local personnel through one-on-one training. Increased supervision will also improve adherence to established program protocols. In Sirajganj, sub-districts with markedly higher STH prevalence warrant attention.

WHO has recommended PC for all non-pregnant WRA “living in areas” with baseline STH prevalence ≥ 20% among WRA [3]. The MOHFW is considering where and how to apply this guidance. In Bangladesh, there are no baseline data for WRA. ICSPM data could fill this need. However, it would require surveying the remaining districts in the country, and the required resources are unavailable. In ICSPM-surveyed districts that have ≥ 20% prevalence among WRA, the MOHFW has a sound basis for expanding administration of PC to this risk group. Given the low prevalence in three of four ICSPM districts, treating 46.6 million WRA *nationwide* is unwarranted. Again, there are important implications for having statistically valid district-level data.

As the MOHFW considers WHO guidance on PC for WRA, notably there was low hookworm prevalence (< 0.7% overall). Hookworm infections are of concern to that risk group because of resulting anemia. While our surveys relied on Kato-Katz testing, with its challenges in detecting hookworm in samples more than a few hours old [16], the lack of hookworm infections is consistent with past surveys in Bangladesh among children [17–21] and WRA [22]. Furthermore, a 2018 systematic review on STH combined the results of seven studies (*n* = 2886) from Bangladesh and estimated prevalence of hookworm to be only 3%. Given the program goal, the lack of hookworm and *T. trichiura* infections means that treating WRA, at least nationwide, would unnecessarily divert resources from the main STH parasite and the population it impacts: *A. lumbricoides* in children [23].

National programs in resource-limited countries, such as Bangladesh, have often relied on school-based parasitologic monitoring of STH. Unlike WHO-recommended school-based surveys, the ICSPM provides the MOHFW the necessary data to understand program status at the implementation unit (district) level. Because the MOHFW relies on *district* public health and education systems to deliver interventions and monitor PC coverage, it follows that assessment of reported PC coverage and STH epidemiology at the district level is useful for program managers. Assessing the impact of control efforts in a district also provides critical guidance to district health and education offices.

### Limitations

For practicality and cost, the surveys were designed to assess STH prevalence by WHO prevalence range to a threshold of < 10%. Notably, data from multiple districts could be combined to reach a sample size that would have adequate statistical power for assessing the ≤  1% MHII goal, but this could result in modifying PC frequency without accounting for heterogeneity in the geographic distribution of STH.

Non-response was almost 40% across surveyed districts. There was a large disparity in participation among adult males and females, both in those enrolled, and those who provided a sample. In future ICSPMs, the MOHFW plans to address high non-response by improving coordination with local health authorities and increasing pre-survey community mobilization. The MOHFW is considering whether it is logistically and financially feasible, during future ICSPMs, to make follow-up visits to relevant households. As discussed, recall bias could have led to inaccurate ICSPM PC coverage estimates. Finally, although participants were asked to provide stool samples in the morning, substantial delays between stool deposit and examination were possible. Such delays may have led to the underestimation of hookworm prevalence.

## Conclusion

The ICSPM provided a needed, innovative approach to assess programmatic progress. The surveys highlighted areas where high prevalence persists, despite 20 MDA rounds, and that require enhanced monitoring and intervention delivery. ICSPMs require more resources than school-based parasitologic surveys; however, the resources used in Bangladesh were comparable to similar surveys (e.g. LF Transmission Assessment Surveys, Trachoma district mapping), and ICSPMs serve multiple purposes and achieve important efficiencies.

Documenting the impact of years of PC will help focus program resources on areas of persistent high transmission, as in Sirajganj. The resources freed up by a geographically-targeted approach could meet other needs including establishing ongoing parasitologic monitoring in low prevalence districts and improving PC coverage among risk groups other than SAC.

## Data Availability

The datasets generated for and analyzed in the study are not publicly available but are available from the corresponding author upon reasonable request.

## Abbreviations

ICSPM: Integrated Community-based Survey for Program Monitoring
LF: Lymphatic filariasis
MDA: Mass drug administration
MHII: Moderate-to-high intensity infection of STH
MOHFW: Ministry of Health & Family Welfare, Government of Bangladesh
PC: Preventive chemotherapy
PSAC: Preschool-age children
SAC: School-age children
STH: Soil-transmitted helminthiasis
WASH: Water, sanitation, and hygiene
WHO: World Health Organization
WRA: Women of reproductive age
